# Cognitive impact of multidomain intervention and omega 3 according to blood Aβ42/40 ratio: a subgroup analysis from the randomized MAPT trial

**DOI:** 10.1186/s13195-023-01325-3

**Published:** 2023-10-23

**Authors:** Julien Delrieu, Bruno Vellas, Sophie Guyonnet, Christelle Cantet, Vitaliy Ovod, Yan Li, James Bollinger, Randall Bateman, Sandrine Andrieu, on behalf of MAPT/DSA group, Isabelle Carrié, Lauréane Brigitte, Catherine Faisant, Françoise Lala, Hélène Villars, Emeline Combrouze, Carole Badufle, Audrey Zueras, Christophe Morin, Gabor Abellan Van Kan, Charlotte Dupuy, Yves Rolland, Céline Caillaud, Pierre-Jean Ousset, Sherry Willis, Sylvie Belleville, Brigitte Gilbert, Francine Fontaine, Jean-François Dartigues, Isabelle Marcet, Fleur Delva, Alexandra Foubert, Sandrine Cerda, Marie-Noëlle-Cuffi, Corinne Costes, Olivier Rouaud, Patrick Manckoundia, Valérie Quipourt, Sophie Marilier, Evelyne Franon, Lawrence Bories, Marie-Laure Pader, Marie-France Basset, Bruno Lapoujade, Valérie Faure, Michael Li Yung Tong, Christine Malick-Loiseau, Evelyne Cazaban-Campistron, Françoise Desclaux, Colette Blatge, Thierry Dantoine, Cécile Laubarie-Mouret, Isabelle Saulnier, Jean-Pierre Clément, Marie-Agnès Picat, Laurence Bernard-Bourzeix, Stéphanie Willebois, Iléana Désormais, Noëlle Cardinaud, Marc Bonnefoy, Pierre Livet, Pascale Rebaudet, Claire Gédéon, Catherine Burdet, Flavien Terracol, Alain Pesce, Stéphanie Roth, Sylvie Chaillou, Sandrine Louchart, Kristel Sudres, Nicolas Lebrun, Nadège Barro-Belaygues, Jacques Touchon, Karim Bennys, Audrey Gabelle, Aurélia Romano, Lynda Touati, Cécilia Marelli, Cécile Pays, Philippe Robert, Franck Le Duff, Claire Gervais, Sébastien Gonfrier, Yannick Gasnier, Serge Bordes, Danièle Begorre, Christian Carpuat, Khaled Khales, Jean-François Lefebvre, Samira Misbah El Idrissi, Pierre Skolil, Jean-Pierre Salles, Nicola Coley

**Affiliations:** 1 Maintain Aging Research team, CERPOP, Université de Toulouse, Inserm, Université Paul Sabatier, Toulouse, France; Gérontopôle, Department of Geriatrics, Toulouse CHU, Toulouse, France; 2 Department of Neurology, Washington University School of Medicine, St. Louis, MO USA; 3 Knight Alzheimer Disease Research Center, Washington University School of Medicine, St. Louis, MO USA; 4 Maintain Aging Research team, CERPOP, Université de Toulouse, Inserm, Université Paul Sabatier, Toulouse, France; Department of Epidemiology and Public Health, Toulouse CHU, Toulouse, France

**Keywords:** Clinical trial, Alzheimer’s disease, Amyloid blood biomarker, Prevention

## Abstract

**Background:**

In MAPT (Multidomain Alzheimer Preventive Trial), a cognitive effect of multidomain intervention (MI) was showed in non-demented subjects with positive amyloid PET. However, screening eligible patients for multidomain intervention by PET is difficult to generalize in real-world settings.

**Methods:**

MAPT study was a 3-year, randomized, placebo-controlled trial followed by a 2-year observational and optional extension. All participants were non-demented and randomly assigned (1:1:1:1) to the MI plus omega 3, MI plus placebo, omega 3 alone, or placebo alone group. The objectives were to assess the cognitive effect of MAPT interventions (omega 3 supplementation, MI, combined intervention) in non-demented subjects according to amyloid blood status at 12, 36, and 60 months. In this subgroup analysis (*n* = 483), amyloid status was defined by plasma Aβ42/40 ratio (cutoff ≤ 0.0107). The primary outcome measure was the change in cognitive composite score after a 1, 3, and 5-year clinical follow-up.

**Results:**

The intention-to-treat (ITT) population included 483 subjects (161 positive and 322 negative amyloid participants based on plasma Aβ42/40 ratio). In the positive amyloid ITT population, we showed a positive effect of MI plus omega 3 on the change in composite cognitive score in 12 (raw *p* = .0350, 0.01917, 95% CI = [0.0136 to 0.3699]) and 36 months (raw *p* = .0357, 0.2818, 95% CI = [0.0190 to 0.5446]). After correction of multiple comparisons and adjustments, these differences were not significant (adjusted *p* = .1144 and .0690). In the per-protocol positive amyloid group (*n* = 154), we observed a significant difference between the combined intervention and placebo groups at 12 (*p* = .0313, 0.2424, 0.0571 to 0.4276) and 36 months (*p* = .0195, 0.3747, 0.1055 to 0.6439) persisting after adjustment. In the ITT and per-protocol analyses, no cognitive effect was observed in the positive and negative amyloid group at 60-month visit.

**Conclusions:**

These findings suggest a benefit of MI plus omega 3 in positive blood amyloid subjects. This promising trend needs to be confirmed before using blood biomarkers for screening in preventive trials.

**Trial registration:**

ClinicalTrials.gov Identifier: NCT01513252.

## Background

The MAPT (Multidomain Alzheimer Prevention Trial) study has tested cognitive effect of omega 3 polyunsaturated fatty acid supplementation (omega 3) and multidomain intervention (MI) in non-demented subjects with memory complaint [1]. In the total population of the MAPT study, MI and omega 3 had no significant effect on cognitive decline over 3 years [2]. Nevertheless, the FINGER (Finnish Geriatric Intervention Study to Prevent Cognitive Impairment and Disability) and MAPT studies showed concordant effects in subgroups of at-risk subjects. In FINGER, the cognitive beneficial effect of the MI was greater than that of the control intervention in APOE ε4 carriers but not in non-carriers [3]. In the ancillary amyloid MAPT study (MAPT-AV45), the MI effect was positive only in non-demented subjects with positive amyloid positron emission tomography (PET) [4]. These findings could suggest cognitive effect of a MI in early stage on the continuum of Alzheimer disease (AD). However, MAPT-AV45 and FINGER studies had several methodological limitations: (1) the long-term impact of MI was not evaluated after interruption of the interventional program to test durability, (2) the sub-group size of MAPT-AV45 was relatively low, and (3) APOE ε4 status used in FINGER to define at-risk subjects for cognitive decline cannot be considered as a diagnosis biomarker of AD. To date, amyloid level assessed by PET and cerebrospinal fluid (CSF) measures of Aβ isoforms are the most widely used amyloid biomarkers. Screening by amyloid PET is difficult to generalize in real-world settings given its cost and limited access. Blood-based biomarkers are less invasive and cost-effective options for identification of at-risk subjects eligible for these non-pharmacological interventions [5]. Recent improvements in technologies used to assess amyloid blood levels have shown promising results [6]. Several groups have showed that the blood Aβ42/40 ratio provides a sensitive and reliable measure of amyloid status, well correlated to CSF Aβ42/40, that can predict future brain amyloidosis (i.e., conversion to positive amyloid PET) [7–9]. These promising results suggest that plasma Aβ42/40 ratio could be used to detect amyloid plaques in individuals before cognitive symptoms onset. However, these markers still need to be validated in interventional studies for the selection of potential participants. In prevention trials, a blood Aβ42/40 test could be used as screening tool to identify at-risk subjects for AD and to facilitate pharmacological and non-pharmacological program discovery [10–12].

In a subgroup of the MAPT study, amyloid blood assays have been performed from the MAPT biobank to determinate amyloid status of the participants. These data are an opportunity to validate encouraging findings from MAPT-AV45 and to assess the possibility of such preventive trials based on blood biomarkers in the future. Moreover, two additional years of clinical observation were performed after completion of the MAPT interventional program to track durability of the intervention once discontinued. Therefore, we evaluated the long-term cognitive effect over a 36-month treatment period and at 60 months, 24 months after discontinuation of non-pharmacological intervention in the subgroup characterized by blood biomarkers.

## Methods

### Study design and participants

**Fig. 1:**
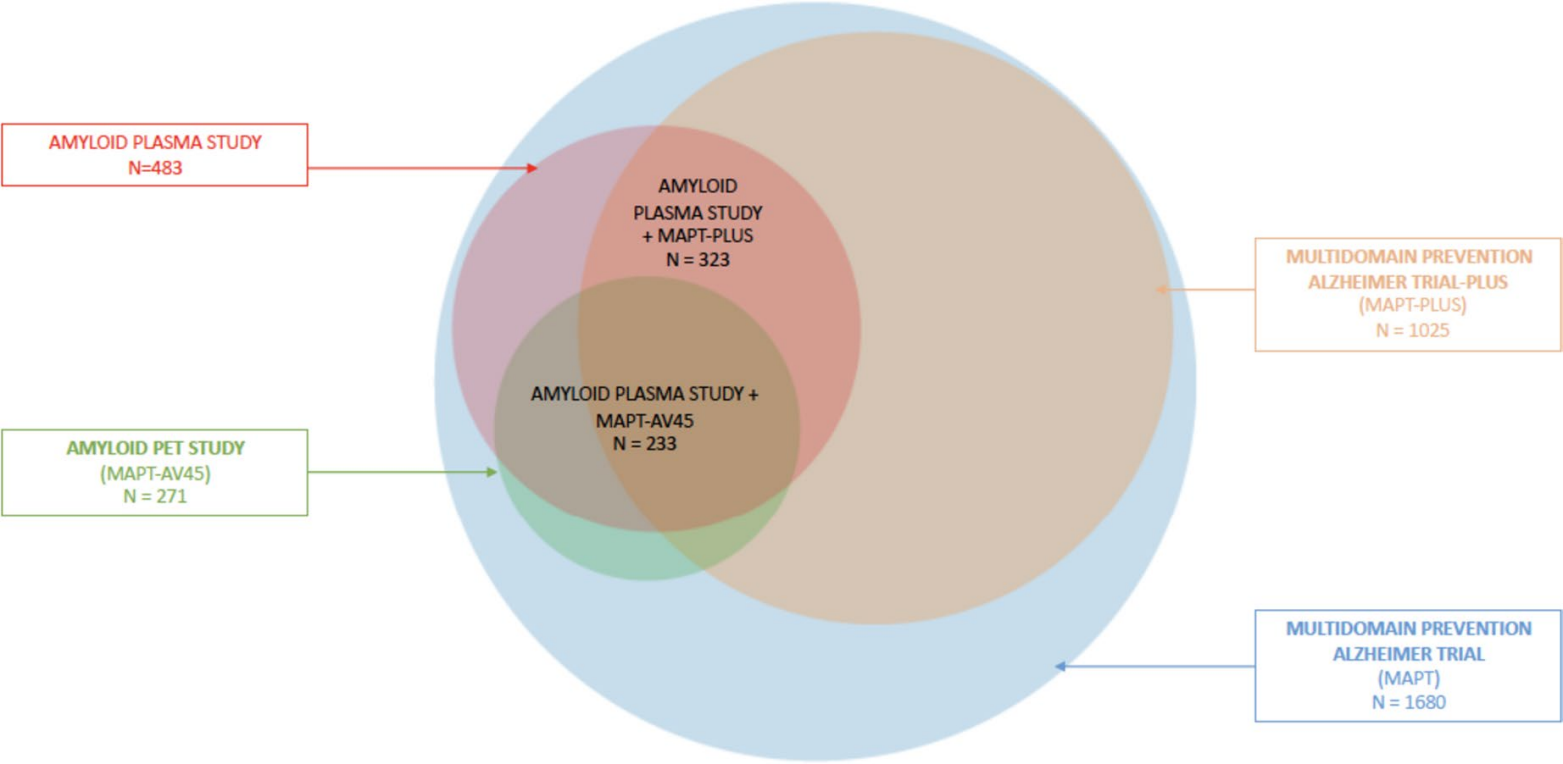
Place of the amyloid plasma analysis in relation to MAPT and ancillary studies. MAPT multidomain Alzheimer’s preventive trial

All subjects included in the present analysis were participants, from the MAPT and MAPT-PLUS studies, that were tested for amyloid blood biomarkers (Fig. 1). MAPT was a multicenter (13 memory centres in France and Monaco), randomized, placebo-controlled, 3-year trial whose objective was to assess effect of MI and omega 3 on cognitive performance. MAPT-PLUS was a 2-year observational and optional extension of MAPT after completion of the interventional program [1]. The objective of MAPT-PLUS was to evaluate the long-term cognitive effect of MAPT preventive strategies. This additional follow-up was systematically offered to MAPT participants during the end-of-study visit.

Based on MAPT inclusion criteria, subjects included in the present analysis were non-demented, 70 years old and over, and fulfilled one of the following three criteria: spontaneous memory complaint, limitation in one instrumental activity of daily living, or slow gait speed [2].

### Randomization and masking

In MAPT, participants were randomly assigned (1:1:1:1) to the MI plus omega 3, MI plus placebo, omega 3 alone, or placebo alone group. A computer-generated randomization procedure was used with block sizes of eight and stratification by center. A clinical research assistant used a centralized interactive voice response system to identify which group to allocate the participant and which lot number to administer [2]. All participants and research staff including neuropsychologists were blinded to omega 3 or placebo assignment and to amyloid blood status.

### Procedures

Participants took two capsules of either the placebo or omega 3 daily. The active supplement used was V0137, an oil mixture containing natural fish oil with a minimum of 65% docosahexaenoic acid (400 mg) and a maximum of 15% eicosapentaenoic acid (no more than 112.5 mg). As described previously, MI program consisted of 12 2-h group sessions focusing on three domains (cognitive stimulation, demonstrations of physical activity, and nutritional advice) and a preventive consultation for the management of cardiovascular risks at baseline, 12 and 24 months [2]. This interventional program lasted 3 years, and 2-year observational follow-up was added in MAPT-PLUS.

Clinical visits in MAPT and MAPT-PLUS were scheduled every 6 or 12 months to assess physical and functional conditions and adherence [1]. Cognitive assessment included the Free and Cued Selective Reminding Test (FCSRT) [13], the Controlled Oral Word Association Test and Category Naming Test (CNT) [14], the Digit Symbol Substitution Subtest of the Wechsler Adult Intelligence Scale–Revised [15], the Trail-Making Tests [16], the Mini-mental State Examination [17], and the Clinical Dementia Rating scale (CDR) [18]. Physical evaluation included the Short Physical Performance Battery (SPPB) [19] and Fried frailty criteria [20]. Autonomy in daily living activities was evaluated by the Alzheimer’s disease Cooperative Study-Activities of Daily Living Prevention Instrument (ADCS-ADL) [21]. One blood sample of 15 ml (10 ml in an EDTA vacutainer and a pair of × 2.5 ml in PAXgene RNA tubes) was collected yearly for the biobank. These samples were transferred directly at ambient temperature to the Cellular Biology and Cytology Laboratory at each site. The two PAXgene RNA tubes were frozen at −20° directly. The EDTA tube was centrifuged then aliquoted; the serum and the pellet were collected in two 5-ml dry tubes, then frozen at −20°. A biobank scientific committee has identified amyloid blood biomarkers as a research priority.

### Plasma Aβ42/Aβ40 immunoprecipitation/mass spectrometry assay methods

Plasma samples of 0.46 ml were assessed to test plasma Aβ_42_ and Aβ_40_ levels by immunoprecipitation mass spectrometry as previously described [9, 22]. Aβ levels were analyzed and calculated by integrated peak area ratios to known concentrations of the internal standards using the Skyline software package [23].

Aβ42/Aβ40 cutoff (≤ 0.0107) has been defined, by Randall Bateman laboratory at Washington University School of Medicine in Saint-Louis, to discriminate as accurately as possible negative and positive amyloid participants in comparison to PET [24]. Indeed, many subjects included in the present analysis (*n* = 233) were participants from MAPT-AV45 with amyloid PET (Fig. 1). In the MAPT-AV45 study, the positivity threshold for amyloid PET was set at SUVr > 1.17 [4].

### Adherence

For omega 3 supplementation and placebo, subjects were considered as adherent if they returned less than 25% of the prescribed capsules. For MI program, participants were considered as adherent if they attended at least 75% of the group sessions (if applicable) [2].

### Primary outcome and objectives

The primary outcome measure was the change in cognitive composite score after a 1, 3, and 5-year follow-up. We used a composite of four measures, close to the PACC (Preclinical Alzheimer Cognitive Composite), well established to show sensitivity to decline in early stages of AD [25]. The MAPT cognitive composite score has been already described previously [2, 4, 26]. This cognitive composite score was calculated by combining FCSRT, CNT, Digit Symbol Substitution Subtest, and MMSE orientation scores.

The main objectives were to assess according to amyloid blood status: (1) the cognitive effect of MAPT interventions at 12 and 36 months and (2) the long-term impact at 60 months after 2-year interruption of these interventions.

### Statistical analysis

Analysis was completed in the intention-to-treat (ITT, *n* = 483) and per-protocol (*n* = 457) populations. The ITT population consisted of all randomly assigned participants who completed a cognitive composite score at baseline and a minimum of one post-baseline visit. Per-protocol population excluded all major protocol violations at baseline and during follow-up [2]. Efficacy in subgroups according to amyloid blood status was assessed by post-hoc analysis.

We used the same statistical method as for the work carried out to determine the cognitive effect of MAPT interventions according to PET amyloid status [4]. Linear mixed-model repeated-measures analyses were used including baseline, 6, 12, 24, 36, 48, and 60-month follow-up data to assess between-group differences in the change in cognitive composite score from baseline to 12, 36, and 60 months. Time was used as a continuous variable, with a cubic trajectory, because the terms time^2^ and time^3^ were significant. For each linear mixed model, we included subject-specific random effects to consider the intra-subject correlation: a random intercept to consider the heterogeneity of the composite score at baseline and a random slope to consider the heterogeneity of the slopes between subjects. In the unadjusted linear mixed models, we included these fixed effects: intervention group by their amyloid blood status (8 categories), time, and interaction between group and time [4]. Then, to test the difference of the effect of the intervention between the negative and positive amyloid blood groups, we used the estimates of the interaction term parameters with the ESTIMATE command from the SAS MIXED procedure.

All the models were completed with and without adjustments for gender, age, educational level, CDR global score, and APOE ε4 genotype. All *p* values were presented before and after adjustment for multiple comparisons (using the Hochberg procedure) and the statistical significance was set at a *P* value < 0.05. All confidence intervals were two-sided with a 95% confidence level. All statistical analyses were achieved using SAS software version 9.4 (SAS Institute Inc, Cary, NC).

### Standard protocol approvals, registrations, and patient consents

The MAPT protocol is listed in a public-access clinical trial database (www.clinicaltrials.gov, no. NCT01513252). Written informed consent was given by all participants. A new informed consent form was signed by participants who volunteer for MAPT-PLUS during the end-of-study visit.

### Data availability

The datasets generated and/or analyzed during this study are not publicly available. However, clinical data can be shared upon request following completion of the MAPT/DSA Data Access Application form (for further information contact the Data Sharing Alzheimer group: Info.u1027-dsa@inserm.fr).

## Results

### Enrollment and rates of study completion

**Fig. 2:**
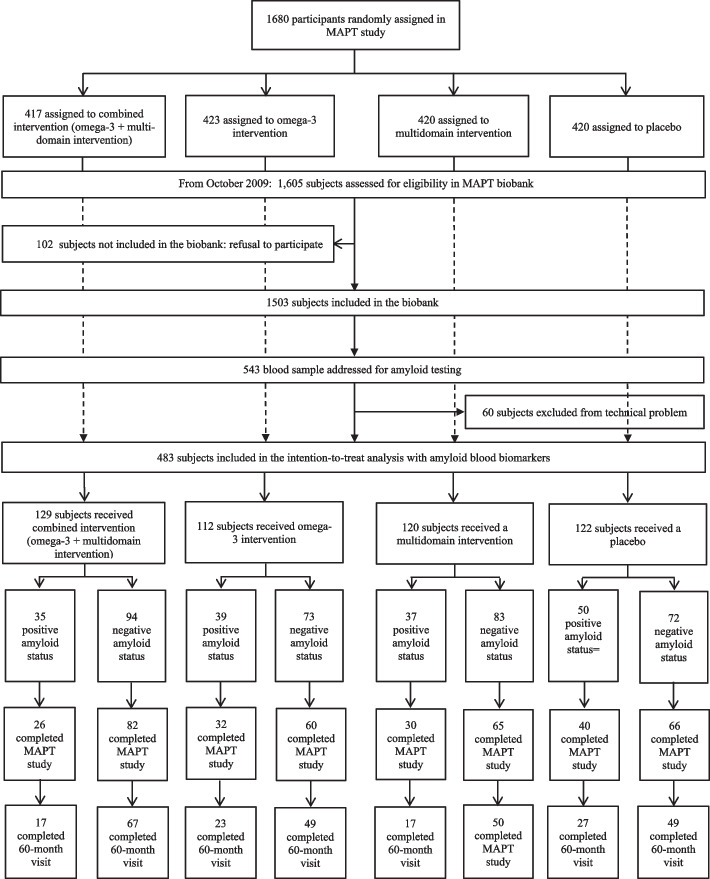
Trial profile for the amyloid blood MAPT study. MAPT multidomain Alzheimer’s preventive trial, MI multidomain intervention

Among the 1680 participants in MAPT and 1503 in its biobank, 483 amyloid blood assays were performed for this analysis at 12 (448 subjects) and 24 months (35 subjects). These subjects (*n* = 483) were selected from MAPT biobank based on their participation in the MAPT-AV45 study and an available blood sample as close as possible to the baseline visit. Subjects were enrolled in the MAPT biobank from October 2009. The mean time interval between blood collection and baseline visit is 12.99 ± 3.15 months. From 483 subjects included in this analysis, 323 subjects had observational data at 48 months and 299 at 60 months in MAPT-PLUS. The flow chart of participants in this analysis is shown in Fig. 2.

The ITT population included 161 positive and 322 negative amyloid subjects based on plasma Aβ42/Aβ40 ratio. In the ITT subgroup with positive amyloid blood status, 128 (79.5%) and 84 (52.2%) subjects completed respectively 36- and 60-month visits. In ITT subgroup with negative blood amyloid status, 273 (84.8%) and 215 (66.8%) subjects completed 36- and 60-month visits.

### Baseline characteristics

Subjects who had amyloid blood assays (*n* = 483) were significantly older (on average 75.78 ± 4.55 vs. 75.15 ± 4.36 years, *p* = 0.0099), more frequently male (40.79 vs. 33.03%, *p* = 0.0026), APOE ε4 carriers (27.63 vs. 20.65%, *p* = 0.0.0047) and compliant to 3-year intervention (68.26 vs. 60.67%, *p* = 0.0045), had more frequently a CDR global score at 0.5 (47.00 vs. 40.08%, *p* = 0.0094), lower cognitive and functional performances respectively in composite cognitive (−0.10 ± 0.69 vs. 0.01 ± 0.67, *p* = 0.0017) and ADCS-ADL scores (39.13 ± 5.08 vs. 39.91 ± 4.66, *p* = 0.0035), than MAPT subjects not included in this analysis (*n* = 1196).

Baseline characteristics (clinical and blood-based biomarkers) of the ITT population are shown in Table 1. In the positive amyloid ITT population, the four groups are different in total SPPB (*p* = 0.0117) but not in the cognitive composite score (*p* = 0.4467, Table 1). In negative amyloid subjects, the four groups are different in plasma Aβ42/40 ratio (*p* = 0.0322) and DHA (*p* = 0.0310) but not in cognitive composite score (*p* = 0.6723, Table 1).

**Table 1:** Baseline characteristics of the intention-to-treat ancillary amyloid blood MAPT study population (*n* = 483)

	ITT population (*n* = 483)							
	**Negative amyloid blood status** (*n* = 322)				**Positive amyloid blood status** (*n* = 161)			
	Omega-3 + MI (*n* = 94)	Omega-3 (*n* = 73)	MI (*n* = 83)	Placebo (*n* = 72)	Omega-3 + MI (*n* = 35)	Omega-3 (*n* = 39)	MI (*n* = 37)	Placebo (*n* = 50)
**Subject characteristics**								
Male gender, *N* (%)	40 (42.55)	27 (36.99)	26 (31.33)	24 (33.33)	20 (57.14)	20 (51.28)	18 (48.65)	22 (44.00)
Age in years, mean (SD)	75.97 (4.77)	75.52 (4.64)	75.08 (4.44)	75.11 (3.96)	76.69 (4.78)	77.23 (4.45)	75.30 (4.52)	76.54 (4.69)
Education, *N* (%)								
No diploma or primary school certificate	18 (19.35)	20 (28.17)	17 (20.99)	21 (29.17)	8 (22.86)	14 (36.84)	11 (29.73)	12 (24.49)
Secondary education	35 (37.63)	19 (26.76)	27 (33.33)	16 (22.22)	14 (40.00)	15 (39.47)	15 (40.54)	17 (34.69)
High-school diploma	16 (17.20)	8 (11.27)	17 (20.99)	12 (16.67)	4 (11.43)	2 (5.26)	2 (5.41)	8 (16.33)
University level	24 (25.81)	24 (33.80)	20 (24.69)	23 (31.94)	9 (25.71)	7 (18.42)	9 (24.32)	12 (24.49)
APOE ε4 carrier, *N* (%)	17 (20.24)	9 (14.06)	16 (21.33)	19 (28.79)	11 (34.38)	12 (32.43)	19 (55.88)	18 (39.13)
DHA (μg/g RBC), mean (SD)	30.08 (7.97)	29.70 (9.43)	32.84 (9.76)	33.34 (10.48)	28.63 (9.69)	32.85 (10.20)	31.24 (10.15)	31.72 (9.43)
Plasma Aβ42/40, mean (SD)	0.12 (0.02)	0.12 (0.01)	0.12 (0.01)	0.12 (0.01)	0.10 (0.01)	0.10 (0.01)	0.10 (0.01)	0.10 (0.01)
**Cognition**								
Cognitive composite score, mean (SD)	0.01 (0.66)	0.09 (0.60)	0.06 (0.68)	0.13 (0.57)	-0.26 (0.90)	-0.10 (0.68)	-0.21 (0.82)	-0.02 (0.57)
MMSE total score/30, mean (SD)	28.27 (1.58)	28.18 (1.61)	27.95 (1.76)	28.21 (1.48)	27.86 (1.54)	27.72 (1.99)	27.84 (1.50)	27.50 (1.64)
MMSE orientation score/10, mean (SD)	9.87 (0.42)	9.88 (0.41)	9.77 (0.55)	9.86 (0.39)	9.57 (0.74)	9.74 (0.55)	9.65 (0.68)	9.82 (0.48)
CDR score, *N* (%)								
CDR = 0	63 (67.02)	38 (52.05)	40 (48.19)	41 (56.94)	16 (45.71)	19 (48.72)	19 (51.35)	20 (40.00)
CDR = 0.5	31 (32.98)	35 (47.95)	43 (51.81)	31 (43.06)	19 (54.29)	20 (51.28)	18 (48.65)	30 (60.00)
FCSRT scores, mean (SD)								
Free recall/48	26.87 (6.76)	27.51 (6.04)	27.24 (7.66)	27.43 (6.22)	25.20 (7.82)	27.33 (6.78)	24.41 (7.76)	25.92 (6.64)
Total recall/48	44.38 (4.33)	45.63 (3.27)	44.80 (4.76)	45.35 (3.41)	43.91 (4.61)	44.87 (4.37)	44.57 (4.01)	44.78 (4.46)
Delayed free recall/16	10.40 (2.83)	10.74 (2.72)	10.47 (3.11)	10.65 (3.00)	9.40 (3.94)	10.21 (3.18)	9.38 (3.62)	10.04 (2.88)
Delayed total recall/16	15.19 (1.53)	15.42 (1.18)	15.29 (1.70)	15.46 (1.06)	14.74 (1.87)	15.36 (1.09)	14.92 (1.46)	15.28 (1.34)
TMT A, mean (SD)	49.87 (17.93)	49.51 (22.23)	46.82 (19.14)	46.18 (17.73)	48.14 (19.74)	47.41 (13.44)	51.11 (20.93)	44.10 (10.56)
TMT B, mean (SD)	128.86 (56.53)	129.40 (72.96)	114.72 (46.86)	112.30 (39.45)	139.94 (104.15)	134.05 (52.82)	131.53 (68.08)	116.32 (40.36)
Code test score, mean (SD)	36.29 (9.58)	35.92 (10.20)	37.81 (10.52)	37.92 (9.32)	35.40 (9.14)	34.28 (9.58)	35.19 (11.58)	36.28 (7.86)
COWAT score, mean (SD)	19.37 (6.22)	19.34 (6.90)	19.47 (6.75)	20.15 (6.68)	19.63 (7.50)	18.72 (5.69)	18.89 (6.11)	18.76 (5.91)
CNT score, mean (SD)	24.78 (7.19)	25.95 (7.27)	26.20 (7.93)	26.15 (6.95)	23.54 (8.40)	24.31 (7.43)	24.08 (7.05)	25.16 (6.72)
**Other measures**								
ADCS-ADL PI /45; mean (SD)	39.16 (4.63)	39.03 (5.26)	39.17 (5.06)	38.54 (6.05)	38.68 (4.35)	39.49 (5.27)	39.51 (5.42)	39.72 (4.35)
GDS, mean (SD)	2.92 (2.41)	3.08 (2.86)	3.57 (2.94)	3.01 (2.88)	2.57 (1.97)	3.23 (2.56)	2.81 (2.15)	3.08 (2.33)
SPPB, mean (SD)	10.50 (1.53)	10.65 (1.72)	10.28 (1.74)	10.59 (1.55)	10.60 (1.63)	10.23 (1.31)	11.11 (1.37)	10.43 (1.65)
3-year adherence ≥ 75%, N (%)	51 (54.26)	58 (84.06)	40 (48.19)	59 (89.39)	19 (55.88)	30 (93.75)	19 (52.78)	38 (82.61)

*ADCS-ADL* Alzheimer’s Disease Cooperative Study–activities of daily living, *CDR* Clinical Dementia Rating score, *CNT* Category Naming Test, *COWAT* Controlled Oral Word Association Test, *DHA* docosahexaenoic acid, *FCSRT* Free and Cued Selective Reminding Test, *GDS* Geriatric Depression Scale, *MMSE* Mini-Mental State Examination, *SPPB* Short Physical Performance Battery, *TMT* Trail Making Test

### Cognitive impact of MAPT interventions at 12-, 36-, and 60-month visits

**Fig. 3:**
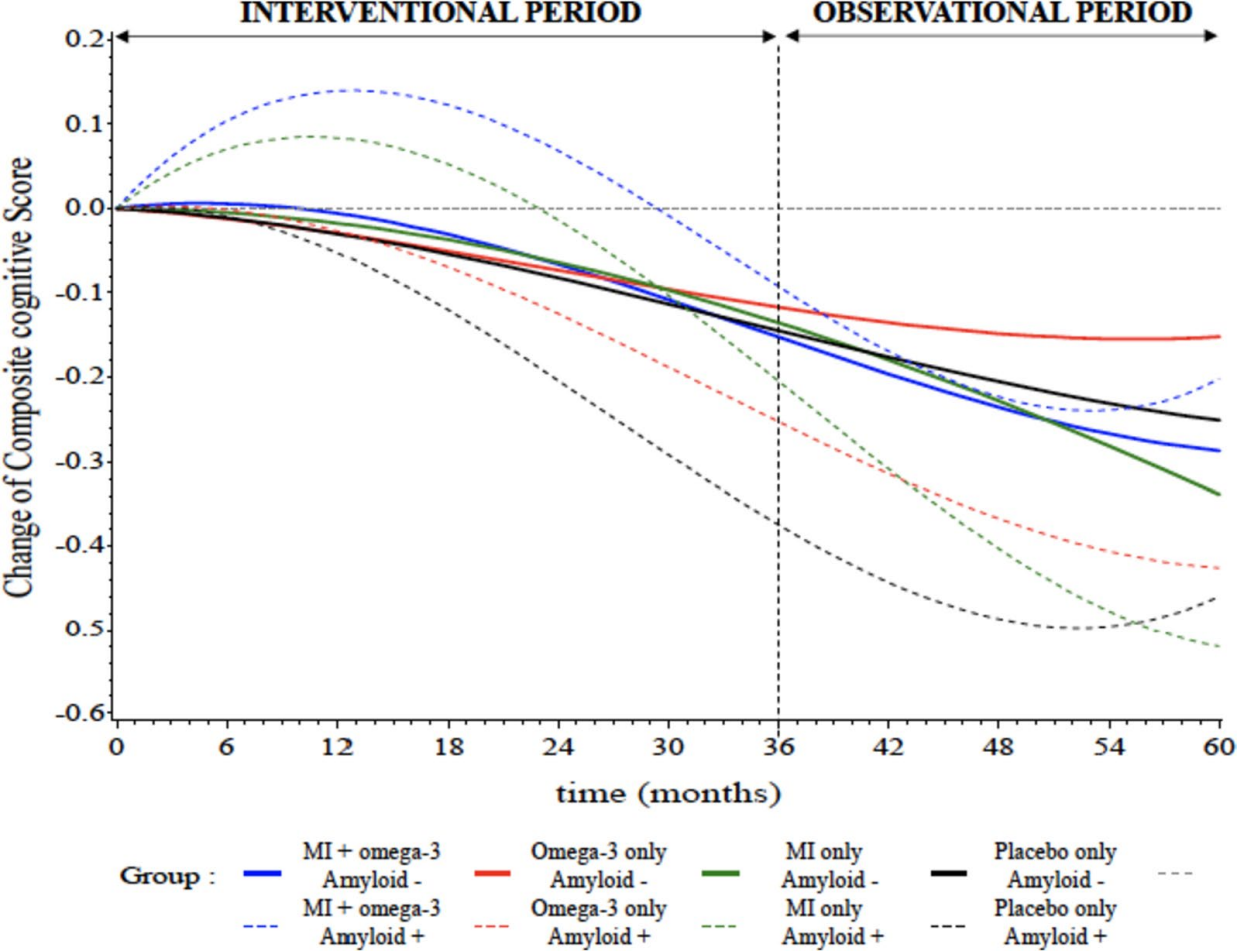
Mean change from baseline in composite cognitive score over 60 months (intention-to-treat population, *n* = 483). MI multidomain intervention, Amyloid+ positive amyloid status, Amyloid − negative amyloid status

The main results are presented in Fig. 3 and Tables 2, 3, and 4.

**Table 2:** Estimated mean difference in 1- and 3-year change from baseline on composite Z score for the three intervention groups compared to the placebo group

**Groups**	**Estimated mean change from baseline (95% CI)**	**Estimated mean between-group difference in change from baseline (95% CI)**					
		**Unadjusted**			**Adjusted**^**a**^		
		**vs. placebo**	**Raw *P***	**Hochberg *P***	**vs. placebo**	**Raw *P***	**Hochberg *P***
**1-year ITT MAPT analysis** (*n* = 483)							
**Positive plasma Aβ42/40**							
Multidomain plus omega 3	0.1392 (0.0018; 0.2765)	0.1917 (0.0136; 0.3699)	**0.0350**	0.1049	0.1891 (0.0104; 0.3679)	**0.0381**	0.1144
Omega 3 alone	-0.0267 (-0.1555; 0.1022)	0.0259 (-0.1458; 0.1976)	0.7669	0.7669	0.0074 (-0.1676; 0.1824)	0.9338	0.9338
Multidomain plus placebo	0.0835 (-0.0490; 0.2160)	0.1361 (-0.0383; 0.3106)	0.1260	0.2519	0.1113 (-0.0649; 0.2874)	0.2153	0.4306
Placebo	-0.0526 (-0.1661; 0.0609)	-	-	-	-	-	-
**Negative plasma Aβ42/40**							
Multidomain plus omega 3	-0.0059 (-0.0884; 0.0765)	0.0241 (-0.1007; 0.1488)	0.7050	0.9950	0.0199 (-0.1066; 0.1463)	0.7578	0.9306
Omega 3 alone	-0.0296 (-0.1240; 0.0648)	0.0004 (-0.1325; 0.1334)	0.9950	0.9950	0.0135 (-0.1584; 0.1879)	0.8445	0.9306
Multidomain plus placebo	-0.0176 (-0.1058; 0.0707)	0.0124 (-0.1162; 0.1411)	0.8494	0.9950	0.0058 (-0.1244; 0.1360)	0.9306	0.9306
Placebo	-0.0300 (-0.1236; 0.0636)	-	-	-	-	-	-
**1-year per-protocol MAPT analysis** (*n* = 457)							
**Positive plasma Aβ42/40**							
Multidomain plus omega 3	0.1880 (0.0440; 0.3319)	0.2435 (0.0582; 0.4287)	**0.0101**	**0.0302**	0.2424 (0.0571; 0.4276)	**0.0104**	**0.0313**
Omega 3 alone	-0.0264 (-0.1564; 0.1036)	0.0291 (-0.1455; 0.2037)	0.7437	0.7437	0.0070 (-0.1705; 0.1845)	0.9383	0.9383
Multidomain plus placebo	0.0846 (-0.0527; 0.2220)	0.1402 (-0.0400; 0.3203)	0.1270	0.2540	0.1132 (-0.0684; 0.2949)	0.2214	0.4428
Placebo	-0.0555 (-0.1721; 0.0611)	-	-	-	-	-	-
**Negative plasma Aβ42/40**							
Multidomain plus omega 3	-0.0022 (-0.0894; 0.0850)	0.0288 (-0.1002; 0.1578)	0.6611	0.8971	0.0229 (-0.1073; 0.1532)	0.7298	0.9902
Omega 3 alone	-0.0399 (-0.1363; 0.0565)	-0.0089 (-0.1443; 0.1265)	0.8971	0.8971	-0.0009 (-0.1375; 0.1358)	0.9902	0.9902
Multidomain plus placebo	-0.0115 (-0.1046; 0.0816)	0.0195 (-0.1136; 0.1526)	0.7735	0.8971	0.0178 (-0.1165; 0.1522)	0.7947	0.9902
Placebo	-0.0310 (0.1261; 0.0641)	-	-	-	-	-	-
**3-year ITT MAPT analysis** (*n* = 483)							
**Positive plasma Aβ42/40**							
Multidomain plus omega 3	-0.0931 (-0.2959; 0.1098)	0.2818 (0.0190; 0.5446)	**0.0357**	0.1071	0.3030 (0.0420; 0.5640)	**0.0230**	0.0690
Omega 3 alone	-0.2530 (-0.4443; -0.0616)	0.1219 (-0.1322; 0.3760)	0.3461	0.3461	0.1257 (-0.1305; 0.3820)	0.3353	0.3353
Multidomain plus placebo	-0.2051 (-0.3972; -0.0130)	0.1698 (-0.0849; 0.4244)	0.1907	0.3461	0.1399 (-0.1148; 0.3946)	0.2808	0.3353
Placebo	-0.3749 (-0.5420; -0.2077)	-	-	-	-	-	-
**Negative plasma Aβ42/40**							
Multidomain plus omega 3	-0.1527 (-0.2729; -0.0325)	-0.0075 (-0.1890; 0.1740)	0.9352	0.9352	-0.0037 (-0.1861; 0.1787)	0.9684	0.9684
Omega 3 alone	-0.1173 (-0.2550; 0.0203)	0.0278 (-0.1657; 0.2213)	0.7775	0.9352	0.0194 (-0.1748; 0.2136)	0.8443	0.9684
Multidomain plus placebo	-0.1356 (-0.2663; -0.0049)	0.0096 (-0.1791; 0.1982)	0.9206	0.9352	0.0180 (-0.1710; 0.2071)	0.8512	0.9684
Placebo	-0.1452 (-0.2812; -0.0091)	-	-	-	-	-	-
**3-year per-protocol MAPT analysis** (*n* = 457)							
**Positive plasma Aβ42/40**							
Multidomain plus omega 3	-0.0298 (-0.2415; 0.1820)	0.3490 (0.0769; 0.6210)	**0.0121**	**0.0362**	0.3747 (0.1055; 0.6439)	**0.0065**	**0.0195**
Omega 3 alone	-0.2534 (-0.4461; -0.0607)	0.1253 (-0.1322; 0.3828)	0.3392	0.3392	0.1254 (-0.1334; 0.3842)	0.3413	0.3413
Multidomain plus placebo	-0.2158 (-0.4142; -0.0173)	0.1630 (-0.0988; 0.4248)	0.2217	0.3392	0.1297 (-0.1317; 0.3911)	0.3299	0.3413
Placebo	-0.3787 (-0.5495; -0.2080)	-	-	-	-	-	-
**Negative plasma Aβ42/40**							
Multidomain plus omega 3	-0.1452 (-0.2725; -0.0179)	0.0047 (-0.1830; 0.1924)	0.9605	0.9605	0.0051 (-0.1827; 0.1930)	0.9572	0.9572
Omega 3 alone	-0.1284 (-0.2683; 0.0116)	0.0216 (-0.1750; 0.2181)	0.8294	0.9605	0.0068 (-0.1897; 0.2033)	0.9457	0.9572
Multidomain plus placebo	-0.1321 (-0.2694; 0.0051)	0.0178 (-0.1768; 0.2123)	0.8576	0.9605	0.0289 (-0.1656; 0.2234)	0.7703	0.9572
Placebo	-0.1499 (-0.2878; -0.0120)	-	-	-	-	-	-

*ITT* intention-to-treat, *MAPT* Multidomain Alzheimer Prevention Trial

^a^Analysis adjusted for age, sex, level of education, APOE ε4 genotype, and clinical dementia rating global score

**Table 3:** Estimated mean difference in 5-year change from baseline on composite Z score for the three intervention groups compared to the placebo group

**Groups**	**Estimated mean change from baseline (95% CI)**	**Estimated mean between-group difference in change from baseline (95% CI)**					
		**Unadjusted**			**Adjusted**^**a**^		
		**vs. placebo**	**Raw *P***	**Hochberg *P***	**vs. placebo**	**Raw *P***	**Hochberg *P***
**5-year ITT MAPT-PLUS analysis** (*n* = 483)							
**Positive plasma Aβ42/40**							
Multidomain plus omega 3	-0.2023 (-0.4824; 0.0778)	0.2575 (-0.1004; 0.6154)	0.1579	0.4737	0.2501 (-0.1071; 0.6073)	0.1691	0.5074
Omega 3 alone	-0.4255 (-0.6705; -0.1804)	0.0343 (-0.2969; 0.3656)	0.8385	0.8385	-0.0288 (-0.3665; 0.3089)	0.8666	0.8666
Multidomain plus placebo	-0.5209 (-0.7967; -0.2451)	-0.0611 (-0.4157; 0.2935)	0.7348	0.8385	-0.1582 (-0.5153; 0.1988)	0.3838	0.7676
Placebo	-0.4598 (-0.6827; -0.2369)	-	-	-	-	-	-
**Negative plasma Aβ42/40**							
Multidomain plus omega 3	-0.2870 (-0.4388; -0.1351)	-0.0358 (-0.2674; 0.1959)	0.7614	0.7614	-0.0237 (-0.2569; 0.2095)	0.8415	0.8415
Omega 3 alone	-0.1524 (-0.3287; 0.0239)	0.0988 (-0.1495; 0.3471)	0.4339	0.7614	0.1008 (-0.1489; 0.3506)	0.4271	0.8415
Multidomain plus placebo	-0.3384 (-0.5079; -0.1690)	-0.0872 (-0.3307; 0.1563)	0.4812	0.7614	-0.1107 (-0.3563; 0.1348)	0.3753	0.8415
Placebo	-0.2512 (-0.4261; -0.0763)	-	-	-	-	-	-
**5-year per-protocol MAPT-PLUS analysis** (*n* = 457)							
**Positive plasma Aβ42/40**							
Multidomain plus omega 3	-0.1315 (-0.4197; 0.1567)	0.3233 (-0.0453; 0.6919)	0.0853	0.2560	0.3202 (-0.0475; 0.6880)	0.0876	0.2628
Omega 3 alone	-0.4254 (-0.6737; -0.1771)	0.0293 (-0.3090; 0.3677)	0.8645	0.8645	-0.0423 (-0.3874; 0.3028)	0.8095	0.8095
Multidomain plus placebo	-0.5349 (-0.8177; -0.2522)	-0.0802 (-0.4445; 0.2842)	0.6652	0.8645	-0.1807 (-0.5482; 0.1868)	0.3340	0.6679
Placebo	-0.4548 (-0.6846; -0.2250)	-	-	-	-	-	-
**Negative plasma Aβ42/40**							
Multidomain plus omega 3	-0.2920 (-0.4524; -0.1315)	-0.0378 (-0.2773; 0.2016)	0.7559	0.7559	-0.0277 (-0.2686; 0.2132)	0.8209	0.8209
Omega 3 alone	-0.1613 (-0.3418; 0.0192)	0.0929 (-0.1605; 0.3462)	0.4710	0.7559	0.0825 (-0.1719; 0.3370)	0.5234	0.8209
Multidomain plus placebo	-0.3388 (-0.5183; -0.1593)	-0.0847 (-0.3373; 0.1679)	0.5098	0.7559	-0.1017 (-0.3570; 0.1536)	0.4334	0.8209
Placebo	-0.2541 (-0.4319; -0.0764)	-	-	-	-	-	-

*ITT* Intention-to-treat, *MAPT* Multidomain Alzheimer Prevention Trial

^a^Analysis adjusted for age, sex, level of education, APOE ε4 genotype, and clinical dementia rating global score

**Table 4:** Estimated mean difference between positive and negative participants in 1-, 3-, and 5-year change from baseline on composite Z score for each intervention group compared to the control group

**Groups**	**Estimated difference between positive and negative subjects for each intervention group (95% CI)**		***P* value**	
	**Unadjusted**	**Adjusted**^**a**^	**Unadjusted**	**Adjusted**^**a**^
**1-year ITT MAPT analysis** (*n* = 483)				
**Multidomain plus omega 3**	0.1677 (-0.0498; 0.3852)	0.1693 (-0.0490; 0.3875)	0.1305	0.1282
**Omega 3 alone**	0.0255 (-0.1916; 0.2427)	-0.0060 (-0.2277; 0.2156)	0.8176	0.9573
**Multidomain plus placebo**	0.1237 (-0.0931; 0.3404)	0.1055 (-0.1145; 0.3255)	0.2629	0.3467
**1-year per-protocol MAPT analysis** (*n* = 457)				
**Multidomain plus omega 3**	0.2147 (-0.0110; 0.4404)	0.2194 (-0.0061; 0.4450)	0.0623	0.0565
**Omega 3 alone**	0.0380 (-0.1830; 0.2590)	0.0079 (-0.2170; 0.2327)	0.7356	0.9453
**Multidomain plus placebo**	0.1207 (-0.1033; 0.3446)	0.0954 (-0.1311; 0.3219)	0.2904	0.4084
**3-year ITT MAPT analysis** (*n* = 483)				
**Multidomain plus omega 3**	0.2893 (-0.0301; 0.6087)	0.3067 (-0.0110; 0.6244)	0.0757	0.0584
**Omega 3 alone**	0.0941 (-0.2253; 0.4135)	0.1063 (-0.2169; 0.4295)	0.5628	0.5181
**Multidomain plus placebo**	0.1602 (-0.1567; 0.4771)	0.1218 (-0.1969; 0.4406)	0.3209	0.4528
**3-year per-protocol MAPT analysis** (*n* = 457)				
**Multidomain plus omega 3**	0.3442 (0.0137; 0.6747)	0.3695 (0.0424; 0.6967)	**0.0413**	**0.0269**
**Omega 3 alone**	0.1038 (-0.2201; 0.4277)	0.1186 (-0.2078; 0.4450)	0.5291	0.4754
**Multidomain plus placebo**	0.1452 (-0.1810; 0.4714)	0.1008 (-0.2261; 0.4277)	0.3819	0.5447
**5-year ITT MAPT-PLUS analysis** (*n* = 483)				
**Multidomain plus omega 3**	0.2932 (-0.1331; 0.7196)	0.2738 (-0.1520; 0.6997)	0.1768	0.2065
**Omega 3 alone**	-0.0645 (-0.4785; 0.3495)	-0.1297 (-0.5534; 0.2940)	0.7592	0.5471
**Multidomain plus placebo**	0.0261 (-0.4040; 0.4563)	-0.0475 (-0.4828; 0.3878)	0.9049	0.8300
**5-year per-protocol MAPT-PLUS analysis** (*n* = 457)				
**Multidomain plus omega 3**	0.3611 (-0.0784; 0.8006)	0.3479 (-0.0903; 0.7861)	0.1069	0.1191
**Omega 3 alone**	-0.0635 (-0.4862; 0.3591)	-0.1248 (-0.5571; 0.3075)	0.7674	0.5701
**Multidomain plus placebo**	0.0045 (-0.4389; 0.4478)	-0.0790 (-0.5277; 0.3697)	0.9841	0.7292

*ITT* Intention-to-treat, *MAPT* Multidomain Alzheimer Prevention Trial

^a^Analysis adjusted for age, sex, level of education, APOE ε4 genotype, and clinical dementia rating global score

#### Positive amyloid group

In the positive amyloid ITT population (*n* = 161), we observed a positive effect of combined interventions (MI plus omega 3) on the change in composite cognitive score in 12 (raw *p* = 0.0350, 0.01917, 95% CI = [0.0136 to 0.3699]) and 36 months (raw *p* = 0.0357, 0.2818, 95% CI = [0.0190 to 0.5446]). After correction of multiple comparisons and adjustments, these differences were not significant (adjusted *p* = 0.1144 and 0.0690). In the per-protocol population (*n* = 154), we showed a significant cognitive effect at 12 (adjusted *p* = 0.0313, 0.2424, 95% CI = [0.0571 to 0.4276]) and 36 months (adjusted *p* = 0.0195, 0.3747, 95% CI = [0.1055 to 0.6439]) in favor of MI plus omega 3 group that persisted after adjustments and correction of multiple comparisons (Table 2). To assess if the interventional effect was durable after 2-year interruption of the interventional program, we tested at 60 months. In both ITT and per-protocol populations, we did not observe a remaining effect at 60 months between the three interventional (MI plus omega 3, omega 3 alone, MI alone) and placebo groups (Table 3).

#### Negative amyloid group

In the ITT and per-protocol populations (respectively *n* = 322 and *n* = 303), no cognitive difference was observed on cognitive composite score change at 12, 36, and 60 months for any of the three interventional groups in comparison to placebo group.

#### Comparison of cognitive impact between negative and positive amyloid subjects

In the ITT population, we showed a non-significant trend in the impact of the MI plus omega 3 on the cognitive composite score at 12 and 36 months for the positive amyloid group in comparison to the negative amyloid group (respectively adjusted *p* = 0.1282/0.0584, 0.1693/0.3067, 95% CI = [−0.0490 to 0.3875]/[−0.0110 to 0.6244]). This difference was significant in the per-protocol population at 36-month visit (adjusted *p* = 0.0269, 0.3695, 95% CI = [0.0424 to 0.6967]). There was no difference for the three interventional groups on cognitive composite score between the positive and negative amyloid groups at 60-month visit (Table 4).

## Discussion

This work suggests a significant benefit of combined interventions at 1 and 3 years only in the amyloid positive group. These effects were significant both in magnitude and statistically in the per protocol population. These findings indicate that future prevention trials could target amyloid positive non-demented individuals for interventions utilizing multi-domains. We have demonstrated the utility of a blood-based biomarker to determine amyloid status of individuals likely to respond to intervention. This could enable future prevention trials to have more rapid screening and to enroll many more positive amyloid participants. The blood-based biomarker also enables prevention trials in regions without access to amyloid PET or CSF analyses. We failed to reach significantly different cognitive effect of a prevention program in non-demented subjects according to amyloid blood status at 5 years, after 2 years off treatment, demonstrating that the intervention effect is not durable after 2-year discontinuation.

Previously, in MAPT-AV45, we showed a cognitive impact of MI at 36 months in subjects with a positive amyloid PET and an association between MI and amyloid burden (lower in participants receiving MI) [4, 27]. Our findings confirm the potential cognitive benefit of non-pharmacological prevention strategies as MI in subjects with early AD. One of the main goals of prevention and precision medicine in AD is to deliver diagnosis and prevention “tailored” to the biological characteristics of cognitive unimpaired individuals [28]. Amyloid PET is proposed to be part of precision medicine [29] but blood-based biomarkers are potentially more cost-efficient and accessible tools in real-world settings and thus could be promising screening exams in a prevention and precision strategy.

### Strengths

The strengths of our ancillary study were the long duration of interventional and observational periods. The implementation of an observational period after completion of interventional program allowed to assess long-term cognitive effect and its potential durability. In our knowledge, this work is the first analysis—to date—that assessed cognitive effect of a non-pharmacological intervention considering amyloid status defined by blood-based biomarkers.

### Limitations

Our study has several limitations. First, the sample size is limited given that 483 subjects were divided into 8 groups. Second, amyloid blood biomarkers were not performed at the baseline visit, but in 12 (*n* = 448) and 24 months (*n* = 35). As in MAPT-AV45 study, we hypothesized that amyloid status does not change during follow-up and the risk of amyloid status misclassification is relatively low marginal in the present analysis [4]. Third, the sensitivity and specificity of the plasma amyloid cutoff (≤ 0.0107) were 43.3% and 79.4% respectively with an area under curve (AUC) of 0.634 in comparison to amyloid PET. This AUC is relatively poor and potentially related to the time interval between blood test and amyloid PET scan. Kappa coefficient was 0.2365 (95% CI = 0.1126–0.3605) between amyloid blood ratio and amyloid PET. Most blood biomarkers were performed at 1-year visit while PET scans were performed all along the MAPT follow-up. Also, it is known that amyloid blood tests become positive about 5 years before amyloid PET scans [9], and this could account for some discrepancy. Another limitation in using blood biomarkers is that the difference in amyloid ratio between positive and negative groups is relatively small (10–15%) potentially due to dilution of Aß from central nervous system to peripheral compartment. Thus, inter-assay variability and accuracy of the measurement may significantly contribute to decrease in AUC. Participants were not blinded to MI. It is possible some of difference between the MI plus omega 3 and placebo was attributable to the fact that participants knew whether or not the MI was given [4, 27]. It is also noted that the analysis of subjects according to amyloid blood status was not pre-specified in the statistical analysis plan and was only exploratory.

## Conclusions

Considering the mentioned limitations, these results show a consistent pattern in favor of a MI effect in positive amyloid subjects. A new model of services in dementia prevention may need to be developed and to update the health offer with more efficient access to blood AD biomarkers and prevention program as MI. Blood biomarkers could offer opportunities to screen non-demented subject in future prevention programs and also detect brain amyloidosis in subjects with memory complaint in primary care [28]. Other blood tests could be evaluated to select subjects eligible for prevention programs. Subjects with a positive ptau blood test have also the potential to respond to prevention programs such as MI. These promising results need to be confirmed in others prevention studies prior their use in prevention trials and general practice [30, 31]. Using blood biomarkers as a tool for cognitive interventions may be valuable and this work may help open that door for future trials.

## Data Availability

The datasets generated and/or analyzed during the current study are not publicly available. However, clinical and blood biomarkers data can be shared by request via « Application for Access to the MAPT Database» (for further information contact of the Data Sharing Alzheimer group: Info.u1027-dsa@inserm.fr).

## Abbreviations

AD: Alzheimer disease
ADCS-ADL: Alzheimer’s disease Cooperative Study-Activities of Daily Living Prevention Instrument
AUC: Area under curve
CDR: Clinical Dementia Rating scale
CNT: Category Naming Test
CSF: Cerebrospinal fluid
DHA: Docosahexaenoic acid
EPA: Eicosapentaenoic acid
FCSRT: Free and Cued Selective Reminding Test
FINGER: Finnish Geriatric Intervention Study to Prevent Cognitive Impairment and Disability
ITT: Intention-to-treat
MI: Multidomain intervention
MAPT: Multidomain Alzheimer Prevention Trial
PACC: Preclinical Alzheimer Cognitive Composite
PET: Positron emission tomography
SPPB: Short Physical Performance Battery
